# Evaluation of Antioxidant Defence Systems and Inflammatory Status in Basketball Elite Athletes

**DOI:** 10.3390/genes14101891

**Published:** 2023-09-29

**Authors:** Alessandro Gentile, Carolina Punziano, Mariella Calvanese, Renato De Falco, Luca Gentile, Giovanni D’Alicandro, Ciro Miele, Filomena Capasso, Raffaela Pero, Cristina Mazzaccara, Barbara Lombardo, Giulia Frisso, Paola Borrelli, Cristina Mennitti, Olga Scudiero, Raffaella Faraonio

**Affiliations:** 1 Department of Molecular Medicine and Medical Biotechnology, University of Naples Federico II, 80131 Naples, Italy; alexgenti98@libero.it (A.G.); carolina.punziano@unina.it (C.P.); mariellacalvanese99@gmail.com (M.C.); ciro.miele1987@gmail.com (C.M.); pero@unina.it (R.P.); cristina.mazzaccara@unina.it (C.M.); barbara.lombardo@unina.it (B.L.); gfrisso@unina.it (G.F.); raffaella.faraonio@unina.it (R.F.); 2 Division of Laboratory Medicine, Istituto Nazionale Tumori—IRCCS Fondazione Pascale, 80129 Naples, Italy; renato.defalco@istitutotumori.na.it; 3 Integrated Department of Laboratory and Transfusion Medicine, University of Naples Federico II, 80131 Naples, Italy; gentilebiotech@gmail.com; 4 Department of Neuroscience and Rehabilitation, Center of Sports Medicine and Disability, AORN, Santobono-Pausillipon, 80122 Naples, Italy; ninodalicandro@libero.it; 5 UOC Laboratory Medicine, Hematology and Laboratory Haemostasis and Special Investigations, AOU Federico II University of Naples, 80131 Naples, Italy; menacapasso@libero.it; 6 CEINGE, Biotecnologie Avanzate s.c.ar.l., 80131 Naples, Italy; 7 Task Force on Microbiome Studies, University of Naples Federico II, 80100 Naples, Italy; 8 Department of Medical, Oral and Biotechnological Sciences, Laboratory of Biostatistics, University G. d’Annunzio of Chieti-Pescara, Via dei Vestini 31, 66100 Chieti, Italy; paola.borrelli@unich.it

**Keywords:** physical activity, athletes, biochemical biomarkers, γ-glutamyl transpeptidase, glutathione, urine, vitamin A, vitamin E, oxidative stress, interleukins

## Abstract

Intense physical activity can induce metabolic changes that modify specific biochemical biomarkers. In this scenario, the purpose of our study was to evaluate how intense physical activity can affect oxidative metabolism. Following this, fifteen professional basketball players and fifteen sedentary controls were recruited and subjected to two samplings of serum and urine in the pre-season (September) and two months after the start of the competitive season (November). Our results have shown an increase in athletes compared to controls in CK and LDH in September (respectively, *p*-value 0.003 and *p*-value < 0.001) and in November (both *p*-value < 0.001), whereas ALT is increased only in November (*p*-value 0.09). GGT serum levels were decreased in athletes compared to controls in both months (in September *p*-value 0.001 and in November *p*-value < 0.001). A gene expression analysis, carried out using *RT-PCR*, has revealed that *IL-2*, *IL-6*, *IL-8*, *xCT* and *GCLM* are increased in athletes in both months (*p*-value < 0.0001), while *IL-10* and *CHAC1* are increased only in September if compared to the controls (respectively, *p*-value 0.040 and *p*-value < 0.001). In conclusion, physical activity creates an adaptation of the systems involved in oxidative metabolism but without causing damage to the liver or kidney. This information could be of help to sports doctors for the prevention of injuries and illnesses in professional athletes for the construction of the athlete’s passport.

## 1. Introduction

It is known that constant physical activity carried out adequately and followed by a correct diet has a positive impact on the physical and mental health of the individual [1,2]. Physical activity can prevent obesity, cardiovascular disease, muscular atrophy and increased fat tissue [3,4,5,6,7,8]. In fact, leisure sport is closely connected with an improvement in the quality of life [9]. However, many studies have shown that intense and prolonged physical activity can determine metabolic adaptations [10], which are represented by alterations occurring in the body in response to the intensity and duration of physical exercise [11,12]. Therefore, these adaptations translate into alterations in the concentration and activity of specific biochemical and hematological parameters, and their identification could represent a new strategy to safeguard the health of an athlete [13,14,15,16]. Serum creatine kinase (CK) and lactate dehydrogenase (LDH) can be considered as indicators of the degree of metabolic adaptation to the physical training of skeletal muscles [17,18,19,20,21]. These enzymes are involved in muscle metabolism, and their low concentration in serum is a result of the physiological wear and tear of the cell [18,21]. After an intense exercise, CK usually increases after 24–36 h, representing an important biomarker for monitoring recovery efficiency [20]. Likewise, an increase in LDH levels has been found after intense exercise and muscle injuries [22].

On the other hand, the liver enzymes alanine aminotransferase (ALT), aspartate aminotransferase (AST) and γ-glutamyl transferase (GGT) were also found to be essential for assessing the athlete’s state of health [23,24]. In athletes, the increase in serum aminotransferase concentration after long-distance exercise, such as an ultra marathon, induces chronic liver injury [25]. In particular, ALT and GGT are considered as specific markers for liver injury, and their levels are increased after long-distance running; the degree of liver injury is related to the intensity and duration of the performance [26].

Furthermore, the main cause of cellular damage after high-intensity exercises is the disruption of the redox balance following excess production of reactive oxygen species (ROS), which causes oxidation and modification of numerous cellular components that perturb homeostasis [27,28]. These chemically reactive molecules represent a set of compounds consisting of unpaired electrons in an external electronic shell with high reactivity [29], generated physiologically by body cells following catabolism/anabolism processes. In a balanced living system, levels of reactive ROS species vary in a regulated fashion and are maintained through the use of antioxidants and other enzymes [30]. However, when this equilibrium is disrupted and ROS levels become unmanageable, oxidative stress becomes evident, resulting in derangements of various cellular and tissue components, including proteins, lipids and Deoxyribonucleic Acid (DNA) [31]. Sports, particularly intense and prolonged physical activities, are one of the stressors implied in the establishment of an oxidative environment due to increased oxygen consumption [32], with several studies highlighting an increase in ROS and Reactive Nitrogen Species (RNS) following aerobic and anaerobic exercise, inducing both acute and chronic effects on athletes and impacting their performance, recovery and overall health [33]. Despite the pivotal role played by exercise in mitigating the development of chronic disease, the cardiovascular system in athletes can be particularly affected when oxidative stress is established. The latter, indeed, is implicated in the development and progression of both inflammation and coagulation that can result in a thrombotic event in both arteries and veins, leading to serious and sometimes life-threatening conditions, such as heart attacks, strokes and deep vein thrombosis (DVT) [34].

A fundamental role in the maintenance of cellular oxidative stress is played by glutathione (GSH) [35,36]. Within cells, total GSH exists free and bound to proteins. Since the enzyme glutathione reductase—which reverts free glutathione from its oxidate form, glutathione disulfide (GSSG)—is constitutively active and inducible upon oxidative stress, free glutathione exists almost exclusively in its reduced form. The ratio of reduced to oxidate glutathione within cells is often used as a marker of cellular toxicity during physical activity [37,38]. 

Solute carrier family 7 member 11 (xCT or *SLC7A11*) encodes the xCT protein, which forms the xc- system with solute carrier family 3 member 2 (4F2hc or SLC3A2). This system is a cysteine/glutamate reverse transporter. *XCT* is highly specific to cystine and glutamate and is responsible for the basic transport activity of the xc- system. Cystine is quickly reduced to cysteine after entering the cell to synthesize endogenous glutathione [39].

In glutathione biosynthesis, the first, rate-limiting reaction is catalyzed by glutamate cysteine ligase (GCL), a heterodimer composed of a catalytic (GCLC) and a modifier (GCLM) subunit. GCLC exhibits all of the catalytic activity of the isolated enzyme and feedback inhibition by GSH. *GCLM* is enzymatically inactive but plays an important regulatory function by lowering the Km of GCL for glutamate and raising the Ki for GSH [40,41].

Glucose-6-phosphate dehydrogenase (*G6PD*) catalyzes the first reaction of the pentose phosphate pathway, involving the conversion of glucose into pentose sugars while providing reduced power in the form of nicotinamide adenine dinucleotide phosphate (NADPH). NADPH plays a pivotal role in the glutathione reductase reaction to recycle the (GSSG) in reduced glutathione (GSH). For this reason, the level of *G6PD* is very important to maintain the antioxidative defense [42,43]. 

*Glutathione*-*specific γ-glutamylcyclotransferase 1* (*CHAC1*) is a protein-coding gene. This gene encodes a member of the γ-glutamylcyclotransferase family of proteins. *CHAC1* has γ-glutamyl cyclotransferase activity toward glutathione catalyzing the cleavage of glutathione into 5-oxo-L-proline and a Cys-Gly dipeptide. It acts specifically on glutathione but not on other γ-glutamyl peptides. For this reason, *CHAC1* may have an important role in the oxidative balance of the cell [44].

Intense and continuous exercise is responsible for activating an inflammatory response, which determines the mobilization and activation of granulocytes, lymphocytes and monocytes, as well as the release of inflammatory factors such as interleukins [10,45,46]. In response to exercise, the first cytokine to increase is interleukin-6 (IL-6), although the extent of the increase will depend on the type, duration and intensity of exercise. In fact, it is known that the concentration of IL-6 increases as much as 100-fold after a marathon race, it is produced locally in contracting skeletal muscles, and the net release from the muscle can account for the exercise-induced increase in arterial concentration [47]. At the same time, the release of IL-6 at the muscle level causes the release of IL-8, implying that the two cytokines work closely in muscle metabolism in athletes [40]. Although it was identified in the 1970s as a growth factor, in recent years, IL-2 has assumed an important role as a mediator of the innate and adaptive response [48]. A study conducted by Kaia evaluated the effect of a four-week exercise program on the secretion of interleukin-2 (IL-2) in elite Taekwondo athletes, recording increased levels of IL-2 [49]. On the other hand, interleukin 10 (IL-10) is a cytokine with potent anti-inflammatory properties when produced during exercise, able to prevent tissue damage [50]. Stress conditions, such as physical exercise, induce an increase in the secretion of IL-6 from muscle contraction, stimulating the release of IL-10. 

Finally, a fundamental role is played by vitamins A and E, considered the main antioxidant vitamins; both protect against the formation of ROS by promoting cell renewal and metabolism useful for maintaining healthy muscle function, especially during the recovery phase after acute physical exercise and endurance [51,52].

Therefore, the aim of our work was to analyze the impact of physical activity on hepatic and renal oxidative metabolism through (a) serum evaluation of muscle and hepatic enzymes (CK, LDH, AST, ALT and GGT); (b) urine analysis; (c) dosage in both serum and in urine of GSH and GSSH; (d) evaluation of the gene expression of *xCT*, *GCLM*, *CHAC1*, *G6PD*, IL-2, IL-6, IL-8 and IL-10 and (e) monitoring of vitamin A and E in serum.

In this study, we have chosen specific biomarkers, involved in muscle, renal and liver metabolism in order to identify significative variations that reflect the body’s adaptions. Through the monitoring of these parameters, training, competition and recovery regimes could be customized, in order to optimize performance, and could also help in the realization of an athlete’s passport.

## 2. Materials and Methods

### 2.1. Study Design

In this study, fifteen professional basketball players and fifteen sedentary controls were recruited. Each person was briefed on the purpose and procedures of the study, and written informed consent was obtained from each subject. Physical characteristics of athletes and controls were age 27 ± 7 years, weight 87 kg ± 11 kg and height 194 cm ± 5 cm as well as age 28 ± 5 years, weight 86 kg ± 8 kg and height 185 cm ± 15 cm. None of the subjects smoked, consumed alcohol or used drugs known to alter chemical parameters. All subjects ate a similar diet throughout the season, and furthermore, team doctors continued to monitor diet during the study. The daily calorie intake is about 3000 calories, specifically: carbohydrates account for about 55–60%, and protein accounts for about 12–15%, while for those who do not exercise, protein accounts for about 10–12% and total lipids about 25–30%, according to recommendations applicable to the general population (LARN, Energy and Nutrient Recommendations for the Italian Population–Italian Society of Human Nutrition ‘96). According to LARN, a person should drink at least 1–1.5 L of water per day and lose as much as possible through sweat, urine, etc. 

The study methods and procedures were approved by the School of Medicine, University of Naples Federico II Ethics Committee (Institutional Review Board (IRB) (200/17)) in accordance with the Declaration of Helsinki 1964 (last update 2013).

### 2.2. Experimental Approach

The players completed the same training program, i.e., they worked out twice a day, with morning sessions consisting of two hours in the gym and afternoon sessions consisting of three hours of basketball practice. This training program was completed every day except for official game days during the season (two games per week). Each game lasts 40 min, divided into 4 quarters of 10 min each. The inclusion criteria are male athletes with 7 years of competitive activity experience. The exclusion criteria are no prior muscle damage, cardiovascular disease or recurrent infections.

### 2.3. Collection Data

Blood, serum and urine samples from each participant were taken at two different stages of the competitive season. The first samples were collected in September (month 0) in the pre-season. The second samples were collected in November (2 months after the start of the season). The same serum and urine samples were taken for the controls. The detection of glutathione (GSH), oxidized glutathione (GSSG), γ-glutamyl transferase (GGT), creatine kinase (CK), lactic dehydrogenase (LDH), aspartate transferase (AST), alanine-transferase (ALT), vitamin A and vitamin E was applied to the serum of the entire study population. Also, GSH and GSSG were monitored in the urine of all participants under study. Finally, gene expression of interleukins (IL-2, IL-6, IL-8 AND IL-10) and stress oxidative genes (*xCT*, *GCLM*, *CHAC1* and *G6PD*) were evaluated in the blood of all participants under study.

### 2.4. Dosage of CK, LDH, AST, ALT and GGT

The blood and urine samples were taken in the morning (8:00 a.m.) before training, after 72 h of rest and 12 h of fasting. As a safeguard measure, all samples were frozen at −80 °C in case any analysis had to be repeated. The serum dosage of CK, LDH, AST, ALT and GGT was evaluated on Architect c16000 through a spectrophotometric method (Creatine Kinase, Lactate Dehydrogenase, Aspartate Aminotransferase, Alanine Aminotransferase and γ-Glutamyl Transferase assays; ABBOTT Diagnostics, USA). All the procedures followed manufacturer’s recommendation.

### 2.5. Urine Evaluation

The urine samples were collected early in the morning and were processed using an automated urine chemistry analyzer (UC3500, Sysmex, Kobe, Japan) and a fluorescence flow cytometer (UF 1000i, Sysmex, Kobe, Japan) according to the instructions of the manufacturer. If necessary, we carried out an optical microscope examination. Instructions were recommended for each subject before urine collection: (1) wash hands thoroughly with soap and water and dry them with a cloth; (2) thoroughly wash the genital organs with soap and water and dry them and (3) eliminate the first flow of urine of the W.C. and collect the second one in the sterile container without interrupting urination. The following parameters were analyzed on urine samples: pH (5.5–7 mg/dL), a specific weight (1005–1030), colour, appearance, presence of bacterial cells (0–1000 n/µL), presence of squamous cells (0–20 n/µL), leukocytes (0–18 n/µL), erythrocytes (0–14 n/µL), proteins (0 e 20 mg/dL), glucose, ketones, bilirubin, haemoglobin, nitrite and leukocyte esterase.

### 2.6. GSH and GSSG Assay

Total GSH levels were evaluated using the Glutathione Colorimetric Detection Kit (Invitrogen). This kit permits the evaluation of the reduced glutathione (GSH) and the oxidated glutathione (GSSG) in two different ways. For the detection of GSH, samples were added with a volume of 5% 5-sulfosalicylic acid, incubated for 10 min at 4 °C and centrifuged at 14,000 rpm for 10 min. Subsequently diluted samples of the supernatants (dilution 1:5) were used for the assay procedure where, following incubation with the Colorimetric Detection Reagent and Reaction Mixture for 20 min at room temperature, GSH was totally recovered in the reduced form, and its concentration was determined by measuring the absorbance at the length of 405 nm with the spectrophotometer Glomax according to the manufacturer’s protocol. The procedure for the detection of GSSG is the same with the only difference being that the samples were also added with 2-vinilpiridine to have the oxidated form of glutathione according to the manufacturer’s protocol.

### 2.7. RNA Extraction and cDNA Synthesis

Ribonucleic acid (RNA) extraction was performed from blood with EDTA (1 mL for each sample) via a Trizol reagent following the manufacturer’s protocol (Life Technologies, Carlsbad, CA, USA). Total RNA was quantified using Nanodrop (ND-1000 UV-Vis spectrophotometer, NanoDrop Technologies, Wilmington, DE, USA) measuring the absorbance at 260 nm and the purity in the ratios 260/280 and 260/230 nm. Reverse transcription of RNA into cDNA then followed; 1000 ng of total RNA was reverse transcribed with the iScriptTM cDNA Synthesis Kit (Bio-Rad, Hercules, CA, USA), according to the manufacturer’s instructions.

### 2.8. Gene Expression using Real-Time PCR

For real-time qPCR experiments, the data from each cDNA sample were normalized using the human housekeeping gene RLP0 (ribosomal protein lateral stalk subunit P0). The specific primers used for the amplification of RLP0, IL-6 and IL-8 were designed based on the nucleotide sequences downloaded via the NCBI database (see Table 1) using Primer3WEB v.4.0.0.

Calculations of relative expression levels were performed using the 2^(−ΔΔCt) method [53], and all analyses were performed in triplicate in order to guarantee the accuracy of results.

### 2.9. Evaluation of Vitamin A and E

Sera were analyzed for vitamin A and E concentrations by means of HPLC procedures using the Agilent 1260 Infinity II (ClinRep^®^ Complete Kit “Vitamins A and E” Recipe, Munich, Germany, UE) according to the manufacturer’s recommendations.

### 2.10. Statistical Analysis

Descriptive analysis was carried out using means and standard deviation (±SD) or median and interquartile range (IQR) for the quantitative variables and absolute and percentage values for the qualitative ones. The Shapiro–Wilk test was applied to assess normality. Univariate comparisons (athletes vs. ctr) were investigated using a non-parametric Wilcoxon rank-sum test for quantitative data. Only for the group of athletes, the non-parametric Wilcoxon matched-pairs signed-rank test was used to compare the differences in the two times considered (0 months and 2 months). Statistical significance was taken at the <0.05 level. All analyses were performed using Stata software v17.0 (StataCorp, College Station, TX 77845, USA).

## 3. Results

### 3.1. Dosage of CK, LDH, AST, ALT and GGT

To monitor how intense physical activity can influence some of the main biochemical parameters involved in hepatic and muscle metabolism, we decided to measure CK, LDH, AST, ALT and GGT. What can be deduced from Table 2 is that at both 0 months and at 2 months, the athletes have significantly increased values compared to the controls. On the other hand, if the athletes are compared to each other, it is noted that only the increase in CK and AST is significant over time.

### 3.2. Urine Analysis

To evaluate how physical activity can affect kidney function, we performed urine analysis. All the parameters analyzed fall within the reference values and show no significant variations (see Table 3), with the exception of bacteria and erythrocytes. Bacteria increase at 0 months in athletes compared with controls and increase at 2 months both when compared with controls and with athletes at 0 months (see Table 3). Erythrocytes, instead, decrease at 0 months in athletes compared with controls and decrease at 2 months both when compared with controls and in athletes at 0 months (see Table 3).

### 3.3. Evaluation of GSH and GSSG Levels

To assess the oxidative stress of competitive athletes, we measured the levels of GSH in both serum and urine (see Table 4 and Table 5). As can be seen from Table 4, athletes in both September (0 months) and November (2 months) show an increase in serum GSH levels compared to controls, and this increase is significant; the opposite is the trend in urine, that is, athletes as shown in Table 5 have lower GSH levels than controls, and even in this case, this reduction is significant.

In addition, we also evaluated the levels of oxidized glutathione (GSSG, Doha, Qatar), which highlighted that there is a decrease in both serum and urine if we compare the athletes with their respective controls and if we compare the athletes with each other.

Finally, we evaluated the ratio between GSH and GSSG and noted an increase in GSH in athletes compared to GSSG in serum samples (Table 4), while we have an increase in GSSG in urine (Table 5).

### 3.4. The Influence of Physical Activity on Gene Expression of IL-2, IL-6, IL-8 and IL-10

**Figure 1:**
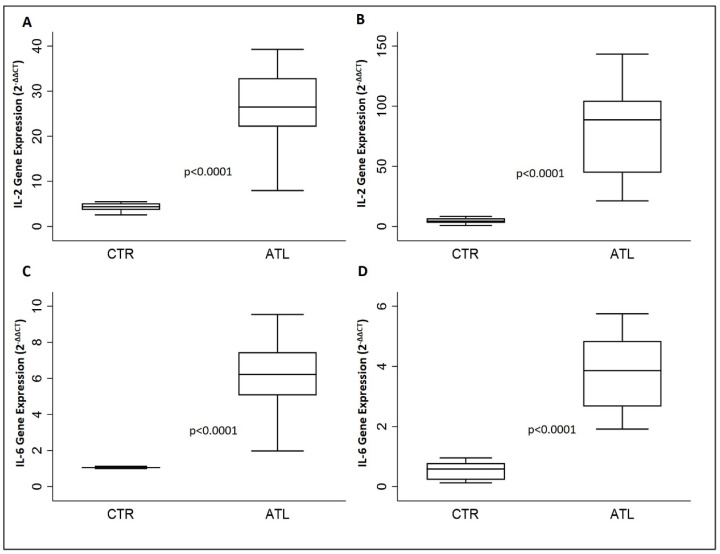
Effect of physical activity on gene expression IL-2 and IL-6 for the two groups compared: IL-2 at month 0 (**A**) and month 2 (**B**); IL-6 at month 0 (**C**) and month 2. (**D**) *p*-values are for Wilcoxon rank-sum test. IL-2, interleukin-2; IL-6, interleukin-6.

**Figure 2:**
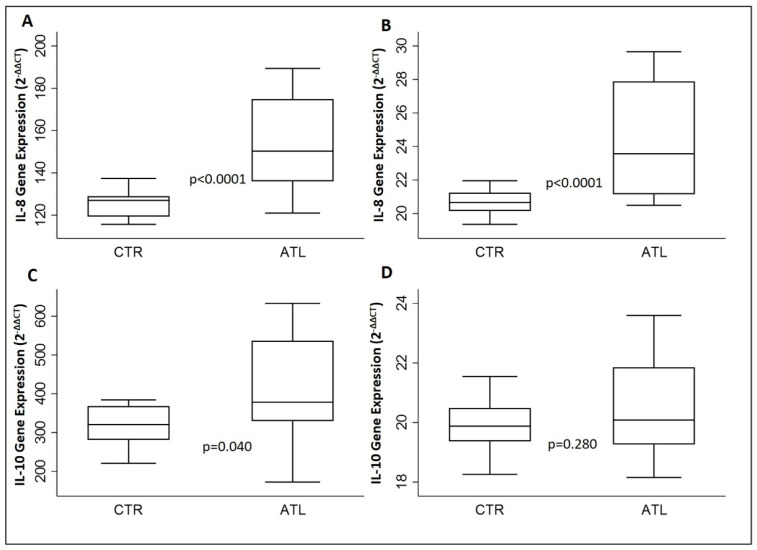
Effect of physical activity on gene expression IL-8 and IL-10 for the two groups compared: IL-8 at month 0 (**A**) and month 2 (**B**); IL-10 at month 0 (**C**) and month 2. (**D**) *p*-values are for Wilcoxon rank-sum test. IL-8, interleukin-8; IL-10, interleukin-10.

To assess if intense physical exercise could modify the gene expression levels of IL-2, IL-6, IL-8 and IL-10 as a result of muscle fatigue, we evaluated the aforementioned interleukins using qPCR (Figure 1 and Figure 2).

Figure 1 shows a significant increase in IL-2 compared to controls in both September (Figure 1A) and in November (Figure 1B). A similar trend is observed for IL-6 when we compare gene expression levels of IL-6 in athletes with the controls in September (Figure 1C) and in November (Figure 1D).

The same results were found regarding IL-8, with an increase in levels in both months if compared to the controls (Figure 2A,B). On the other hand, IL-10 is significantly higher in athletes if compared to the controls in month 0, but in month 2, the difference is not significant, although the same trend is reported (Figure 2C,D).

In addition, we assessed whether intense physical exercise caused changes in interleukin gene expression levels, analyzed exclusively in the athletic population in the two months of September and November. The results are shown in Table 6.

### 3.5. Effects of Physical Activity on xCT, GCLM, CHAC1 and G6PD

**Figure 3:**
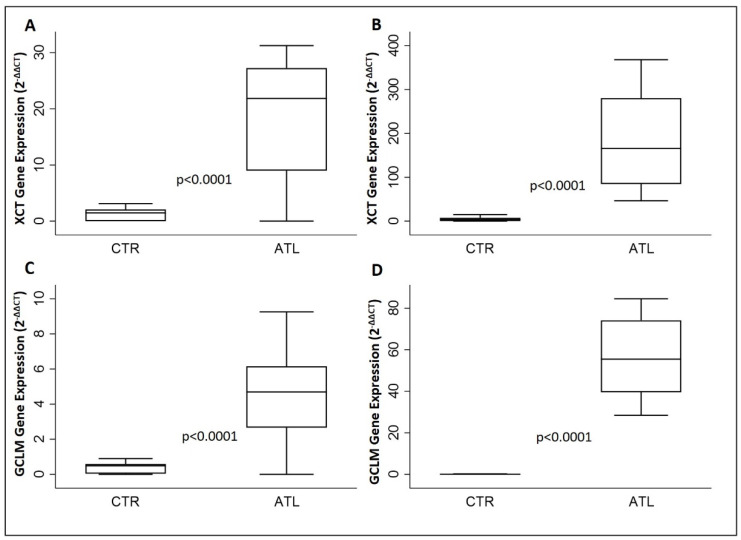
Evaluation of gene expression of XCT and GCLM for the two groups compared: *XCT* in September (**A**) and in November (**B**); *GCLM* in September (**C**) and in November. (**D**) *p*-values are for Wilcoxon rank-sum test. *xCT*, solute carrier family 7 member 11; *GCLM*, glutamate cysteine ligase modifier subunit.

To determine the effect of intense physical exercise on the oxidative stress in athletes compared to controls, we evaluated the gene expression levels of *XCT*, *GCLM*, *CHAC1* and *G6PD*. As reported in Figure 3, *xCT* is increased significantly in the athletic population in comparison to the controls in both months, September and November (Figure 3A,B); the same result has been found in the case of *GCLM* (Figure 3C,D).

**Figure 4:**
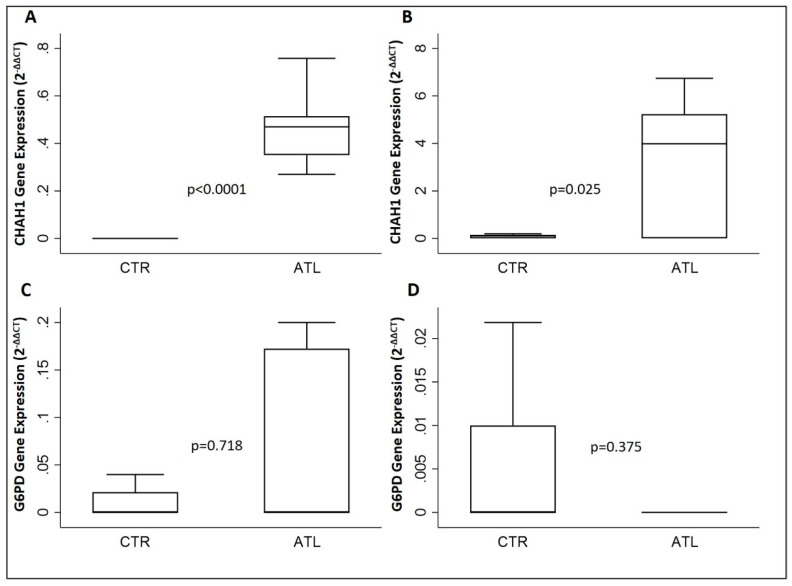
Evaluation of gene expression of CHAC1 and G6PD for the two groups compared: *CHAC1* in September (**A**) and in November (**B**); *G6PD* in September (**C**) and in November. (**D**) *p*-values are for Wilcoxon rank-sum test. *CHAC1*, glutathione-specific γ-glutamylcyclotransferase 1; *G6PD*, glucose-6-phosphate dehydrogenase.

Moreover, we have evaluated the gene expression levels of *CHAC1* and *G6PD*. The results in our possession (Figure 4A,B) show a significant increase in the levels of gene expression of *CHAC1* in September and November, while on the other hand, there is no significant variation for *G6PD* (see Figure 4C,D) in both months.

Finally, we assessed if intense physical exercise caused changes in the gene expression levels *XCT*, *GCLM, CHAC1* and *G6PD* in the athletic population during the months of September and November. The results are reported in Table 7.

### 3.6. The Impact of Physical Exercise on Vitamins A and E

**Figure 5:**
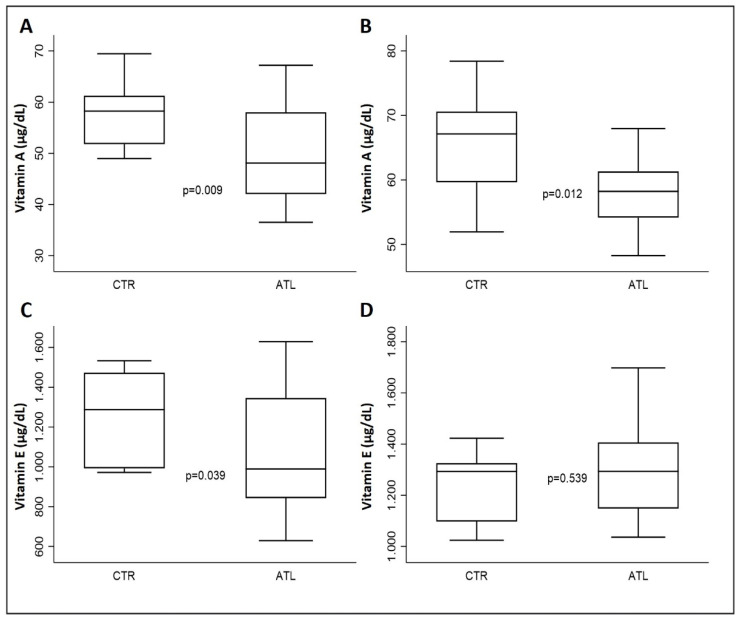
Effect of physical exercise on serum levels of vitamins A and E in the two groups compared: vitamin A in September (**A**) and in November (**B**); vitamin E in September (**C**) and in November. (**D**) *p*-values are for Wilcoxon rank-sum test.

To check the health status, therefore the nutrition and hydration of the agonist athlete, we measured the levels of vitamins A and E in the serum. As can be seen from graph A of Figure 5, the serum levels of vitamin A in both September and November are significantly higher than in the controls; similarly, in graph B of Figure 5, September vitamin E levels are higher in the controls when compared with athletes; on the other hand, in November, the athletes show an increase in the levels of this micronutrient compared to the controls.

## 4. Discussion

In recent years, laboratory medicine has assumed a key role in sports and athlete health monitoring [26]. Numerous studies have shown that intense and continue physical exercise can cause metabolic adaptations, altering the serum concentrations of many biochemical parameters [13,14,15]. The fluctuations of these parameters are an indication of the changes that occur in relation to the intensity and duration of physical exercise as well as to the oxidative stress to which the muscle units are subjected [27]. Following that, we focused our research on the identification of serological, urinary and molecular parameters that would allow us to monitor the health of the competitive athlete, in particular, to monitor the redox and inflammatory status caused by physical activity. 

Our results have shown an increase in CK and LDH in athletes compared to control levels in September and in November; on the other hand, ALT is increased only in November, and GGT serum levels were decreased in athletes compared to controls in both months. Gene expression analysis has revealed that IL-2, IL-6, IL-8, *XCT* and *GCLM* are increased in athletes in both months, while IL-10 and *CHAC1* are increased only in September if compared to controls.

Numerous studies have shown that a persistent and constant increase in CK can induce an alteration in LDH. Therefore, it is necessary to monitor both enzymes in agonists to assess muscle health [17,18,20]. Our athletes are certainly slightly tired, most likely from the load caused by athletic training, which after two months still has to be completely disposed of. In fact, basketball is a sport that involves alternating phases of anaerobiosis and aerobiosis, and during athletic training, there are load phases (muscular endurance); it is known that endurance [54] and aerobic exercise [55] promote the changes of CK and LDH that come with muscle damage after a training session. The magnitude of these changes appears to be related to endurance exercise (RE) protocol [56,57], downhill running [56] and training status [58]. Our results are in line with data obtained from a work conducted in Greece, in which a group of athletes was compared with one of non-athletes, to assess the effect of physical activity on CK levels. This study showed that athletes have increased baseline CK levels compared to non-athletes. It is important to monitor the levels of this parameter since an increase of 5-times higher than the maximum limit exposes the athlete to the risk of exertional rhabdomyolysis (ER) [59].

At the same time, we evaluated the liver enzymes; on the one hand, we noticed an increase in AST at month 2, according to previous studies [22]. The increase in AST occurs in conjunction with the increase in CK and LDH. When the muscle is damaged, such as in response to exercise, it can be released, and its concentration increases in the blood. In fact, although more present in the liver, AST is also present in other districts such as muscle, heart, kidneys, red blood cells, brain and small intestine. Since muscle has a larger tissue mass, it has more AST than that in the liver. For this reason, during muscle disorders or injuries such as those related to vigorous exercise, transaminase levels may be elevated [22,23]. On the other hand, ALT and GGT are not increased in athletes; on the contrary, their concentrations are always lower than in controls and in the normal range. GGT has been proposed as a reliable marker capable of distinguishing liver damage from muscle damage [60]. Therefore, our athletes certainly do not have hepatic suffering caused by physical exercise, and also, with the increase in AST, which can only be seen in time, one should not suggest severe muscle damage such as rhabdomyolysis. In fact, it is common knowledge that hepatic malfunction can cause the manifestation of rhabdomyolysis in participants subjected to physical exercise [61]. 

To assess whether intense physical exercise could have affected renal metabolism, we performed urine analysis. Through this routine methodology, we have seen that the athletes do not have evident variations with respect to the controls, except for some parameters. In this case, we underlined an increase in ketones at time zero, certainly due to diet; in fact, at the time zero before the start of the competitive season, the athletes were returning from holidays, and an unbalanced diet could certainly alter the ketone cycle [62], so much so that the value is normal at the second sampling. Currently, we noticed a slight change in pH and an increase in bacteria, leukocytes and erythrocytes. It is known that athletes have slight variations in pH, which show incorrect hydration also often due to inadequate reintegration of the minerals lost due to intense physical activity [13]; these alterations also facilitate the appearance of bacterial populations, and consequently, there is an increase in the population of lymphocytes and erythrocytes [13]. The values found in the population under study are values that fall within the normal range and certainly do not make us think of kidney damage.

Then, we evaluated the GSH and GSSH levels in both serum and urine in order to assess whether the reductive oxide metabolism of the athletes was not compromised. According to the literature, our results show an increase in GSH in the serum since it certainly supports how physical activity can promote an accumulation of ROS, so athletes produce more GSH than sedentary controls to counteract this accumulation [63]. GGT is the key enzyme in the metabolism of GSH [64], and the results obtained tell us that at the serum level, the GGT decreases and the GSH increases in the opposite trend at the urinary level [65]. Therefore, the glutathione metabolism is certainly functioning correctly and helps athletes to counteract the stress caused by physical exercise [65]. 

It has been shown that intense physical exercise induces numerous intramyocellular signals whose up-regulation lasts from seconds to hours after the end of each training session. These signals eventually result in altered gene expression profiles related to training-induced stress. On this basis, we have evaluated the gene expression of genes involved in oxidative stress. Our results have showed increased levels in *XCT* and *GCLM*, the main genes involved in the biosynthesis of glutathione. In fact, *XCT* is an antiport that allows the entry into the cell of cysteine, one of the constituent amino acids of glutathione, and *GCLM* encodes a subunit of the enzyme glutamate–cysteine ligase necessary for glutathione biosynthesis. On the other hand, the incremented levels of *CHAC1* could be explained as a physiological mechanism linked to increased glutathione levels, since *CHAC1* is one of the genes responsible for the degradation of GSH. Furthermore, we evaluated four important cytokines with gene expression, such as IL-2, IL-6, IL-8 and IL-10. It is known that during intense physical activity, there is an increase in interleukin levels, which work synergy [46], a fact also found in our population that shows this increase is not due to the presence of an infection in progress but rather to an adaptation of the immune system due to the intensity and duration of physical effort [46]. Our results, in accordance with previous studies, show that their increase is constant over time, synonymous with the athlete’s physiological adaptation. Based on our current knowledge, our study is one of the few that chose to assess the impact of exercise on gene expression in elite athletes. Therefore, given the scarcity of results reported in the literature, it is necessary in the future to investigate this aspect in order to identify the main genes that altered their expression in response to intense physical activity.

Finally, we measured the serum levels of two important vitamins with antioxidative capacity: vitamins A and E [51,52]. The levels of both have increased among athletes, an interesting fact that demonstrates that the diet applied is adequate and that their serum increase certainly helps to balance the muscular oxidative stress caused by physical activity [1,2]. Vitamin E, together with GSH, contributes to the inhibition of lipid peroxidation, thus providing support to human health [66].

In the light of this, it is possible to affirm that intense physical activity leads to oscillations of key enzymes of muscle metabolism. At the same time, the athlete’s physiology puts a series of biological and molecular mechanisms in place to counteract fatigue, such as the increase in GSH, which serves to balance the stress caused by physical activity [67,68]. Consequently, GSH and GGT levels could represent “new” liver and kidney biomarkers to monitor and protect the athlete’s health. Therefore, both GSH and GGT must be added to a biomarker panel that has been emerging in recent years and which aims to support sports medicine in constructing ad hoc cures and treatments for each individual athlete [11,13,14,15]. 

In view of the presented results, our study has some limitations. First, the small number of participants. This analysis is a preliminary study, and in future studies, we will recruit more subjects in order to improve the power of the results.

## 5. Conclusions

The biomarkers analyzed in this study are intended to assess the physical condition of the athlete in order to safeguard the health of the athlete by preventing the deterioration of performance due to oxidative stress caused by intense physical activity [27]. This study underlines how a multidisciplinary approach based on biochemical and molecular analysis can support the construction of a panel of specific biomarkers that can be applied to individual athletes. The results obtained would guarantee prevention against the onset of hepatic, musculoskeletal and renal pathologies for each athlete, allowing prompt and targeted treatment by sports doctors.

In conclusion, laboratory medicine represents a valid support for sports medicine by playing a central role in sports and in monitoring the health of athletes [14,26].

## Figures and Tables

**Table 1:** NCBI accession numbers of the genes analyzed.

Gene	Accession Numbers	Primer Forward	Primer Reverse
*RLP0*	NM_053275.4	5′-TGGCAGCATCTACAACCCTG-3′	5′-GACAAGGCCAGGACTCGTTT-3′
*IL-2*	NM_000586.4	5′-AACCTCAACTCCTGCCACAA-3′	5′-GCATCCTGGTGAGTTTGGGA-3′
*IL-6*	NM_001318095.2	5′-CATCCTCGACGG-CATCTCAG-3′	5′-TCACCAGGCAAGTCTCCTCA-3
*IL-8*	NM_001354840.3	5′-AAACCCAGGTGAGAGCTG-3′	5′-TCTGAGATCCCGTCAGAGC-3′
*IL-10*	NM_001382624.1	5′-TCCATTCCAAGCCTGACCAC-3′	5′-AATCCCTCCGAGACACTGGA-3′
*xCT*	NM_014331.4	5′-TGAAATCCCTGAACTTGCGAT-3′	5′-TCTGGATCCGGGCGCT-3′
*GCLM*	NM_002061.4	5′-GACAAAACACAGTTGGAACAGC-3′	5′-CAGTCAAATCTGGTGGCATC-3′
*CHAC1*	NM_024111.6	5′-TTCTGGCAGGGAGACACCTT-3′	5′-GCCTCTCGCACATTCAGGTA–3′
*G6PD*	NM_001360016.2	5′-ACATGAATGCCCTCCACCTG-3′	5′-GGTAGTGGTCGATGCGGTAG-3′

*RPLP0*, ribosomal protein lateral stalk subunit P0; *IL-2*, interleukin-2; *IL-6*, interleukin-6; *IL-8*, interleukin-8; *IL-10*, interleukin-10; *xCT*, solute carrier family 7 member 11; *GCLM*, glutamate cysteine ligase modifier subunit; *CHAC1*, glutathione-specific γ-glutamylcyclotransferase 1 and *G6PD*, glucose-6-phosphate dehydrogenase.

**Table 2:** Biochemical parameters. The data are expressed as the median (IQR). The significance was determined with the Wilcoxon rank-sum test ^§^ or with the Wilcoxon matched-pairs signed-rank test *.

Parameters	Ctr (0 Months)	Athletes (0 Months)	*p*-Value (Ctr vs. 0)	Ctr (2 Months)	Athletes (2 Months)	*p*-Value ^§^ (Ctr vs. 2)	*p*-Value * (0 vs. 2 Months for Athletes)
CK (30–200 U/L) *	138.0 (112.0–158.0)	254.0 (141.0–375.0)	0.003	129.0 (112.0–148.0)	869.0 (551.0–1045.0)	<0.001	<0.001
LDH (125–243 U/L) *	159.0 (148.0–187.0)	208.0 (186.0–257.0)	<0.001	175.0 (148.0–210.0)	267.0 (226.0–302.0)	<0.001	0.053
AST (0–34 U/L) *	21.0 (17.0–28.0)	28.0 (21.0–33.0)	0.048	23.0 (19.0–26.0)	39.0 (34.0–45.0)	<0.001	<0.001
ALT (0–55 U/L) *	34.0 (17.0–42.0)	22.0 (12.0–31.0)	0.094	32.0 (20.0–43.0)	27.0 (21.0–34.0)	0.090	0.037
GGT (12–64 U/L) *	28.0 (19.0–36.0)	15.0 (13.0–19.0)	0.001	36.0 (26.0–41.0)	17.0 (15.0–19.0)	<0.001	0.568

* *Reference values of the Italian Society of Clinical Biochemistry and Clinical Molecular Biology (SIBioC).* CK, creatine kinase; LDH, lactate dehydrogenase; AST, aspartate aminotransferase; ALT, alanine aminotransferase and GGT, γ-glutamyl transferase.

**Table 3:** Urine analysis. The data are expressed as the median (IQR).

Parameters	Value	Ctr (0 Months)	Athletes (0 Months)	*p*-Value (Ctr vs. 0)	Ctr (2 Months)	Athletes (2 Months)	*p*-Value (Ctr vs. 2)
Glucose	Absent	Absent	Absent		Absent	Absent	
Ketones	Negative	Negative	Negative		Negative	Negative	
Bilirubin	Absent	Absent	Absent		Absent	Absent	
Hemoglobin	Absent	Absent	Absent		Absent	Absent	
Nitrite	Absent	Absent	Absent		Absent	Absent	
Leukocyte esterase	Absent	Absent	Absent		Absent	Absent	
Urobilinogen	≤1.0 mg/dL	0.2 (0.2–0.2)	0.2 (0.2–0.2)	1.000	0.2 (0.2–0.2)	0.2 (0.2–0.2)	1.000
Proteins	Absent	Absent	Absent		Absent	Absent	
pH	5.5–7.0 mg/dL	5.5 (5.5–6.0)	6.00 (5.5–6.5)	0.514	5.5 (5.5–6.0)	6.00 (5.5–6.5)	0.015
Bacteria	0–1000 n/µL	1.0 (1.0–3.0)	12.0 (6.0–19.0)	<0.001	7.0 (2.0–9.0)	13.0 (4.0–29.0)	0.006
Leucocytes	0–18 n/µL	4.0 (3.0–8.0)	5.0 (4.0–9.0)	0.438	6.0 (4.0–9.0)	7.0 (3.0–9.0)	0.910
Erythrocytes	0–14 n/µL	5.0 (3.0–9.0)	3.0 (1.0–7.0)	0.052	6.0 (4.0–8.0)	2.0 (0.0–3.0)	<0.001

*Reference values of the Italian Society of Clinical Biochemistry and Clinical Molecular Biology (SIBioC)*.

**Table 4:** Oxidized and reduced serum levels of glutathione. The data are expressed as the median (IQR). The significance was determined with the Wilcoxon rank-sum test ^§^ or with the Wilcoxon matched-pairs signed-rank test *.

Parameters	Ctr (0 Months)	Athletes (0 Months)	*p*-Value (Ctr vs. 0)	Ctr (2 Months)	Athletes (2 Months)	*p*-Value ^§^ (Ctr vs. 2)	*p*-Value * (0 vs. 2 Months for Athletes)
GSH (µM/µL)	0.3 (0.3–0.4)	0.8 (0.5–1.6)	<0.001	0.3 (0.3–0.4)	0.8 (0.5–0.9)	<0.001	0.330
GSSG (µM/µL)	0.1 (0.1–0.1)	0.1 (0.1–0.2)	0.774	0.2 (0.2–0.2)	0.1 (0.1–0.2)	0.002	0.359
GSH/GSSG (µM/µL)	2.6 (2.2–2.9)	6.8 (3.1–13.5)	0.001	1.9 (1.6–2.2)	6.2 (4.4–10.2)	<0.001	0.890

GSH, glutathione; GSSG, glutathione disulfide.

**Table 5:** Oxidized and reduced urine levels of glutathione. The data are expressed as the median (IQR). The significance was determined with the Wilcoxon rank-sum test ^§^ or with the Wilcoxon matched-pairs signed-rank test *.

Parameters	Ctr (0 Months)	Athletes (0 Months)	*p*-Value (Ctr vs. 0)	Ctr (2 Months)	Athletes (2 Months)	*p*-Value ^§^ (Ctr vs. 2)	*p*-Value * (0 vs. 2 Months for Athletes)
GSH (µM/µL)	5.2 (4.2–6.6)	3.7 (3.2–4.2)	0.002	5.7 (4.2–9.1)	3.9 (3.5–4.7)	0.003	0.761
GSSG (µM/µL)	0.9 (0.8–1.0)	0.7 (0.5–0.9)	0.026	0.6 (0.5–0.6)	0.6 (0.5–0.7)	0.566	0.083
GSH/GSSG (µM/µL)	5.8 (5.3–7.7)	5.1 (4.0–7.5)	0.232	11.1 (8.2–16.0)	6.5 (5.9–7.9)	<0.001	0.168

GSH, glutathione; GSSG, glutathione disulfide.

**Table 6:** Comparison of gene expression levels of interleukins in athletic population. The data are expressed as the median (IQR). The significance was determined with the Wilcoxon matched-pairs signed-rank test.

Gene	0 Months	2 Months	*p*-Value
*IL-2*	26.5 (22.1–32.9)	88.6 (44.6–104.6)	<0.0001
*IL-6*	6.2 (5.1–7.5)	3.9 (2.7–4.8)	<0.0001
*IL-8*	150.3 (136.0–174.9)	23.6 (21.2–27.9)	<0.0001
*IL-10*	378.2 (329.3–536.5)	20.1 (19.3–21.9	<0.0001

**Table 7:** Evaluation of gene expression levels of *XCT, GCLM, CHAC1* and *G6PD* in athletic population in month 0 compared to month 2. The data are expressed as the median (IQR). The significance was determined with the Wilcoxon matched-pairs signed-rank test.

Gene	0 Months	2 Months	*p*-Value
*XCT*	21.9 (9.0–27.3)	165.6 (84.5–280.3)	<0.0001
*GCLM*	4.7 (2.7–6.2)	55.5 (39.6–74.2)	<0.0001
*CHAC1*	0.5 (0.4–0.5)	4.0 (0.0–5.2)	0.007
*G6PD*	0.0 (0.0–0.2)	0.0 (0.0–0.0)	0.062

*xCT*, solute carrier family 7 member 11; *GCLM*, glutamate cysteine ligase modifier subunit; *CHAC1*, glutathione-specific γ-glutamylcyclotransferase 1 and *G6PD*, Glucose-6-phosphate dehydrogenase.
